# Salvianolic acid B targets mortalin and inhibits the migration and invasion of hepatocellular carcinoma via the RECK/STAT3 pathway

**DOI:** 10.1186/s12935-021-02367-z

**Published:** 2021-12-07

**Authors:** Mengying Teng, Chunyan Hu, Bingmo Yang, Wei Xiao, Qian Zhou, Yuan Li, Zhong Li

**Affiliations:** The Key Laboratory of Modern Toxicology of Ministry of Education, School of Public Health, Nanjing Medical University, Nanjing, 211166 China

**Keywords:** Hepatocellular carcinoma, Mortalin, RECK, Salvianolic acid B, MMPs, Invasion and migration

## Abstract

**Background:**

Tumor migration and invasion is a complex and diverse process that involves the epithelial–mesenchymal transition (EMT) of tumor cells and degradation of the extracellular matrix by matrix metalloproteases (MMPs). Mortalin is an important oncogene. It has been reported to play an important role in tumor migration and invasion through various signaling pathways, but the underlying mechanism is not fully understood.

**Methods:**

Here, we investigated the role of mortalin in the migration of the hepatocellular carcinoma (HCC) cell lines HepG2 and HCCLM3.

**Results:**

The overexpression of mortalin in HepG2 cells decreased the protein level of reversion-inducing cysteine-rich protein with Kazal motifs (RECK) and activated the phosphorylation and acetylation of STAT3, thereby up-regulating matrix metalloproteinase 9 (MMP9) and promoting cell migration and invasion. In contrast, in HCCLM3 cells, mortalin knockdown increased the expression of RECK, inhibited the STAT3 pathway and the activity of MMP9, and inhibited cell migration and invasion. Furthermore, we found that salvianolic acid B, a caffeic acid phenethyl ester analog, specifically bound to mortalin and increased the degradation of mortalin proteasomes through ubiquitination, thereby up-regulating RECK, inhibiting STAT3, and finally inhibiting the migration and invasion of HCC cells.

**Conclusion:**

Our work suggested that mortalin is a potential therapeutic target for hepatocellular carcinoma.

**Supplementary Information:**

The online version contains supplementary material available at 10.1186/s12935-021-02367-z.

## Background

Hepatocellular carcinoma (HCC) is currently the third leading cause of cancer-related death worldwide [20]. The main treatment of HCC is surgical resection and liver transplantation [3]. However, HCC has a low 5-year survival rate, which is mainly due to a high postoperative recurrence rate and high migration and invasion rates [12]. Therefore, the identification of new therapeutic strategies for HCC is urgent.

Mortalin (mtHsp70), a member of the Hsp70 protein family, is highly expressed in many cancers. Studies have shown that overexpression of mortalin increases the malignant degree of breast cancer cells, and promotes their invasion and metastasis through the PI3K/AKT or JAK/STAT signaling pathway [14]. More importantly, as mortalin is associated with the metastasis of hepatocellular carcinoma, it is considered a tumor marker for predicting early recurrence [27]. At present, the mechanisms by which mortalin promotes migration and invasion have not been studied in detail. According to previous research, the classic way for mortalin to prevent the occurrence and development of cancer is by inhibiting the expression and function of p53 [15]. However, studies have found that the probability of p53 mutation is very high in HCC patients in China [8]. Therefore, there must be other mechanisms by which mortalin affects the occurrence and development of HCC that remain to be explored.

Reversion-inducing cysteine-rich protein with Kazal motifs (RECK) is a glycoprotein located on the cell membrane, and its expression is reduced in tumor cells. Restoration of the RECK levels in malignant cells can inhibit migratory and invasive activities mainly by reducing the expression of the matrix metalloproteinases (MMPs), especially MMP2, MMP9, and MT1-MMP [16]. Walsh et al. showed that RECK can control breast cancer metastasis by modulating the STAT3-dependent neoangiogenic switch [25]. Moreover, STAT3 can regulate MMP2 and MMP9 [6, 29]. In the present study, we have shown that mortalin promoted the migration and invasion of HCC cell lines by regulating the RECK/STAT3 signaling pathway: mortalin down-regulated the expression of RECK protein, and the down-regulation of mortalin led to the up-regulation of RECK protein, which in turn led to the down-regulation of the downstream STAT3 and MMP2/MMP9 signaling pathway. The inhibition of the STAT3 and MMP2/MMP9 signaling pathway reversed EMT and inhibited the migration and invasion of hepatocellular carcinoma cells. We also found that salvianolic acid B (Sal B) inhibited the downstream RECK/STAT3 pathway by targeting mortalin, and finally inhibited the migration and invasion of HCC.

## Materials and methods

### Reagents and cell culture

Sal B (purity ≥ 98%) was purchased from Spring and Autumn Biological Co., Ltd. (China), dissolved in absolute ethanol to a stock concentration of 50 mM, and stored at − 80 °C. Human HCC cell lines, HepG2 and HCCLM3, were purchased from the Institute of Basic Medical Sciences of the China Science Academy, Shanghai. HCCLM3 cells were maintained in high glucose Dulbecco’s Modified Eagle’s Medium (DMEM) and HepG2 cells were maintained in Modified Eagle’s Medium (MEM) supplemented with 10% fetal bovine serum, 100 U/mL penicillin, and 100 mg/mL streptomycin. HepG2 and HCCLM3 cells were grown in the presence of 5% CO_2_ at 37 °C.

### Determination of cell viability

The HCCLM3 cells (2 × 10^4^ cells) were inoculated into 96 well plates for 24 h, and then treated with 0, 100, 200, 300, 400, 500, or 600 μM Sal B for 24 or 48 h. After the treatment, the culture solution containing Sal B was removed and replaced with 100 μL of cell culture medium containing 10% Cell Counting Kit-8 reagent (CCK-8; Dojindo Molecular Technologies, Inc., Kumamoto, Japan) for 4 h. Absorbance at 450 nm was detected using a multi-well plate reader (Model 680; Bio-Rad; Hercules, CA, USA). Untreated cells were used as controls.

### Quantitative real-time polymerase chain reaction (qRT-PCR)

Total RNA was isolated from the cells (after drug treatment of 48 h) that underwent different treatments using TRIzol (Invitrogen, Carlsbad, CA, USA) according to the manufacturer’s recommendations. All primers were synthesized by RiboBio Co. (Guangzhou, China), and the primer sequences are shown in Additional file 1: Table S1. Total RNA (2 µg) was transcribed into cDNA using AMV Reverse Transcriptase (Promega, Madison, WI) for the detection of mRNAs. The reverse transcription procedure was as follows: 25 °C for 10 min, 42 °C for 15 min, 85 °C for 5 min, followed by chilling on ice. The newly synthesized first-strand cDNA was ready for immediate downstream applications or for long-term storage at − 20 °C. The qRT-PCR assays were performed using the LightCycler 96 SYBR Green I Master Mix (Roche) for 40 cycles of 95 °C for 300 s, 95 °C for 10 s, and 60 °C for 30 s. It was carried out with the SYBR Green master mix (Vazyme Biotech Co., Ltd).

### Western blot analysis and immunoprecipitation

Total protein was extracted (after drug treatment of 48 h) using the radio-immunoprecipitation assay (RIPA) buffer (Beyotime Co. Ltd.). A bicinchoninic acid (BCA) kit (Beyotime Co. Ltd.) was used to measure protein concentration. Proteins (20 μg) were separated by 10% sodium dodecyl sulfate-polyacrylamide gel electrophoresis followed by transfer to polyvinylidene fluoride membranes (PVDF; Millipore, Billerica, MA, USA). Antibodies used were RECK, mortalin, E-cadherin, N-cadherin, p-STAT3^Tyr705^, Ac-STAT3^K685^, vimentin (Cell Signaling Technology, 1:1,000), GAPDH and β-actin (Beyotime Co. Ltd., 1:500) (Additional file 1: Table S2). Optical density quantitative analysis was performed using Image-Pro-Plus 6.0 software, and β-actin served as the internal control to correct for differences in protein loading. After the internal reference was normalized, the gray scale ratio compared with the control group was added to the western blot band to observe the trend in molecular changes more objectively.

For immunoprecipitation (IP), the cell protein was extracted and placed on ice for 30 min. After centrifugation, the protein concentration was determined. Cell lysates for IP were incubated with the corresponding antibodies (dilution 1:100) at 4 °C overnight. 50 µL of protein G magnetic beads (PureProteome™, Millipore) were crosslinked with the antibodies (Cell Signaling Technology), and incubated for another 12 h at 4 °C. The immunocomplexes were washed 3 times with TBS-T (0.1%), and bound protein was eluted in 60 µL 0.2 M glycine-HCl (pH 2.5) and neutralized using 5 µL of 1 M Tris (pH 8.5). Eluates were then used to probe an antibody array and subjected to SDS-PAGE and western blotting analysis for mortalin (Cell Signaling Technology) and RECK (Cell Signaling Technology).

### Scratch assay

HepG2 and HCCLM3 cells (5 × 10^5^ cells/well) were seeded in 6-well plates and cultured in DMEM (containing 5% FBS) for 24 h. We used a Thermo Fisher Scientific 10-µL pipette tip to create a scratch wound and then washed the cells with PBS three times, followed by replacement of the DMEM with 0.0–200.0 μM Sal B. Cells were viewed and photographed under a phase-contrast microscope (Olympus) in the same locations at 0, 24 h (for HCCLM3), or 48 h (for HepG2).

### Migration and invasion assays

Transwell assays were performed (after drug treatment of 48 h) using a growth factor reduced Matrigel-coated filter and a non-Matrigel-coated filter (8-mm aperture, BD, Franklin Lakes, NJ, USA) in a 24-well plate. The treated cells were separately trypsinized and inoculated into the upper chamber (5 × 10^4^ cells/well) of the Transwells in serum-free MEM and DMEM, and the lower chamber culture of the Transwells contained 15% FBS. Cells were cultured at 37 °C for 48 h (Matrigel-coated filter) and 24 h (non-Matrigel-coated filter). We used three parallel holes of each experiment for data analysis and five fields of view were randomly selected using an inverted microscope to record the images. The cell count quantitative analysis was performed by Image-Pro-Plus 6.0 software.

### Gelatin zymography

After removing the treatment drug (48 h) and the complete medium, HepG2 and HCCLM3 cells were cultured in a medium containing 1% FBS in MEM and DMEM, and the culture supernatant was collected. Protein concentrations were measured after centrifugation. Proteins previously mixed with non-deformed non-reducing SDS were isolated under non-reducing conditions by an SDS-PAGE (10%) gel containing 1 mg/mL gelatin (Sigma-Aldrich). The separation gel was washed three times with 2.5% Triton X-100/50 mM Tris-HCl (pH 7.6) for 30 min. Then, the gel was incubated in 0.15 M NaCl/10 mM CaCl_2_/50 mM Tris–HCl (pH 7.6/0.05% NaN_3_) for 2 h at 37 °C in a CO_2_-free incubator. The gel was stained with 0.005% Coomassie Blue R250 for 3 h, and decolorized with 10% acetic acid and 10% isopropanol. MMP2 and MMP9 were detected as an effective band on the slab gel.

### Cell transfection

The siRNAs were purchased from Santa Cruz Biotechnology (Additional file 1: Table S3). HCCLM3 cells were seeded in a dish at a density of 5 × 10^5^ cells/well for 24 h, then transiently transfected with Lipofectamine 2000 (Invitrogen) and siRNA for 8 h according to the manufacturer’s instructions. After transfection, the cells were incubated for an additional 24 h in fresh medium with 10% FBS after removal of the medium and then used in other experiments.

The mortalin-Flag plasmid overexpressing both mortalin and Flag was created by inserting the coding sequences of mortalin into the plRES2-3FLAG-EGFP plasmid (Genechem, Shanghai, China). HepG2 cells were seeded in 6-well plates at a density of 1 × 10^5^ cells/well for 24 h, then transiently transfected with vector control (12 ng/mL) or mortalin (12 ng/mL) using Lipofectamine 2000 (Invitrogen) for 12 h according to the manufacturer’s instructions. The medium was then replaced with fresh medium (10% FBS without penicillin–streptomycin), and the treated cells were used for other experiments.

### Docking studies

SYBYL-X software was utilized for molecular-docking of caffeic acid and its derivatives with mortalin. The crystal structure of mortalin [PDB ID: 4KBO] was obtained from PDB (http://www.rcsb.org/pdb/home/home.do). All ligands and water molecules were first removed, and polar hydrogen atoms and AMBER7FF99 charge were added. According to the reported binding region of mortalin and p53 in the literature, the binding sites of CAPE and mortalin are Try196, Phe250, Asp251, Thr267, Asn268, Gly269, Asp270, and Pe272. The residues in the receptors were designated to produce protoplasts using this residue pattern.

### Immunoprecipitation combined with ultra-high-performance liquid chromatography mass spectrometry

The cells of logarithmic HCCLM3 were subcultured and treated with the proteasome inhibitor MG132 for 2 h when the cell convergence reached 60–70%. Cells were cultured for 24 h after replacement of the medium with 100 μM Sal B. Then the cell protein was collected for immunoprecipitation. A total of 100 μL sterilized double-distilled water were added to the precipitate of the control group and the treatment group, and then each mixture was boiled in a protein dry thermostat at 100 °C for 10 min to precipitate the denatured protein, and the tested substance was separated from the protein and dissolved in water for UHPLC/MS analysis. Sal B standard as the positive control, sterile double-distilled water as the control, and PBS and the cell lysate as the samples were to be tested.

The UHPLC/MS analysis used a Thermo Fisher Scientific q active four-stage rod-electrostatic field orbital trap high resolution mass spectrometry system and Dionex UltiMate 3000 high-performance liquid chromatography system. Thermo Fisher Scientific Xcaliber and Thermo Fisher Scientific Pathfinder were used for data collection and processing, respectively.

### Statistical analysis

Data sets were compared using GraphPad-6.0 (GraphPad Software, Inc, La Jolla, CA, USA). Values are presented as the mean ± SD. The difference between two groups was analyzed using a two-tailed Student’s *t*-test. *P* < 0.05 was considered statistically significant.

## Results

### Mortalin promotes the migration and invasion of hepatocellular carcinoma cells

**Fig. 1:**
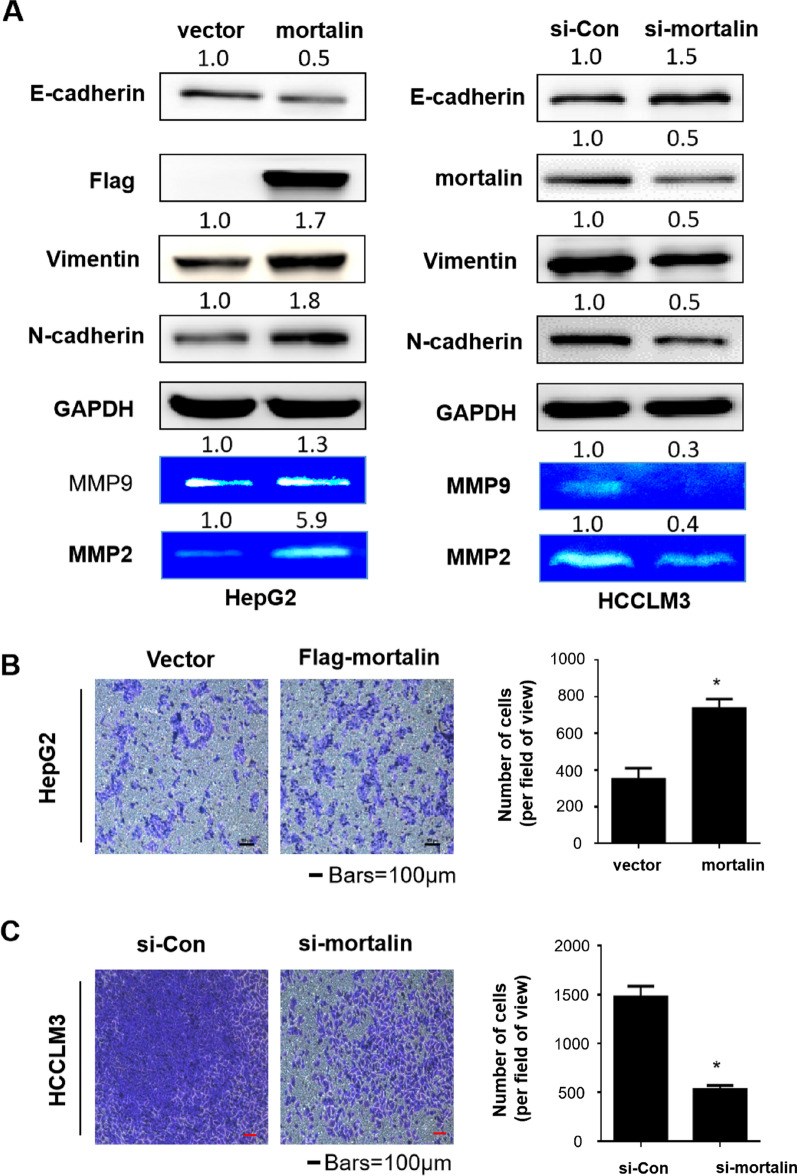
Mortalin can promote the migration of hepatocellular carcinoma cells. **A** Western blot analyses of the expressions of E-cadherin, vimentin, N-cadherin, mortalin, Flag, and GAPDH in HCC cells with different mortalin levels. MMP2 and MMP9 activities were investigated by gelatin zymography assays. **B** HepG2 cells were subjected to the migration assays after overexpression of mortalin, and migrated cells were counted with Stat Monitor in Photoshop (mean ± SD, n = 3). ^*^P < 0.05, statistically significant difference vs. untreated cells. Bars = 100 μm. **C** HCCLM3 cells were subjected to the migration assays after mortalin knockdown with siRNA, and migrated cells were counted with Stat Monitor in Photoshop (mean ± SD, n = 3). ^*^P < 0.05, statistically significant difference vs. untreated cells. Bars = 100 μm

It has been reported that mortalin enhances the process of EMT in breast cancer cells [14]. To further determine the effect of mortalin in HCC, we knocked down mortalin with siRNA or overexpressed mortalin with the viral plasmid Flag-mortalin (Additional file 1: Fig. S4). HCCLM3 is a highly metastatic hepatocellular carcinoma, and its metastatic potential is significantly higher than that of HepG2 cells with low metastatic potential [5]. The expression of mortalin in HCCLM3 was significantly higher than that in HepG2 cells [2]. To explore whether mortalin affects the migration and invasion of HCC cells, we knocked down the mortalin protein in highly invasive HCCLM3 cells and highly expressed the mortalin protein in low invasive HepG2 cells. As shown in Fig. 1A, after mortalin was overexpressed in HepG2, EMT increased, demonstrating that E-cadherin was down-regulated, while N-cadherin and vimentin were up-regulated, whereas knocking-down mortalin in HCCLM3 had the opposite effect, leading to the up-regulation of E-cadherin and down-regulation of N-cadherin and vimentin. In addition, after mortalin was overexpressed in HepG2, the activities of MMPs increased, while knocking-down mortalin in HCCLM3 decreased the activities of MMPs. The Transwell assays confirmed that mortalin overexpression increased cell migration ability in HepG2 (Fig. 1B), while mortalin knockdown in HCCLM3 decreased cell migration ability (Fig. 1C). These results suggest that mortalin promoted the EMT process, the activities of MMPs, and cell migration of HCC.

### Mortalin promotes the migration of hepatocellular carcinoma cells via the RECK/STAT3 signaling pathway

**Fig. 2:**
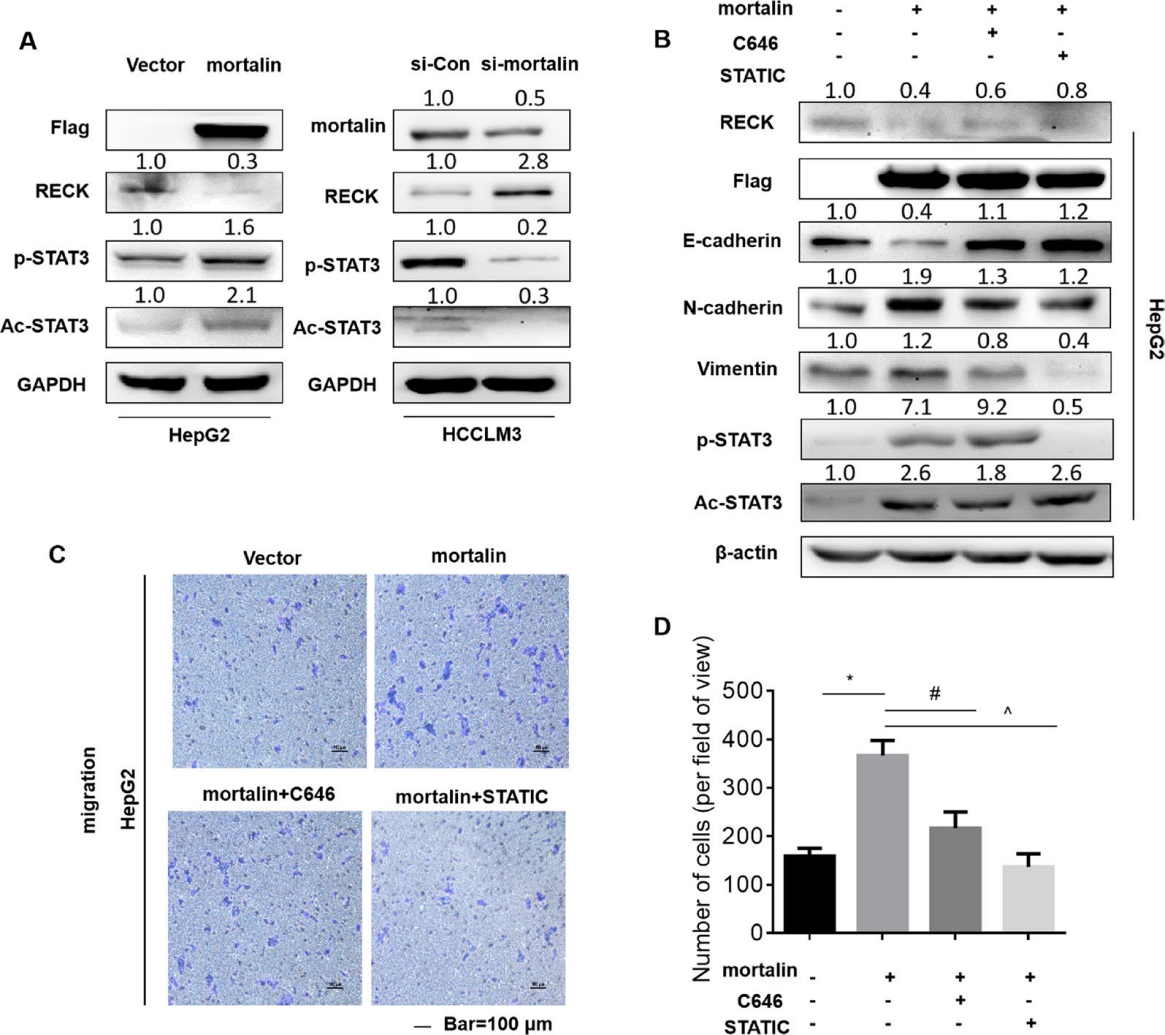
Mortalin regulates the RECK/STAT3 signaling pathway to promote the migration of hepatocellular carcinoma cells. **A** Western blot analyses of the expressions of mortalin, RECK, Flag-mortalin, p-STAT3, Ac-STAT3, and GAPDH in HCC cells with different mortalin levels. **B** Western blot assays of RECK, Flag, E-cadherin, N-cadherin, vimentin, and β-actin were performed. **C** HepG2 cells were subjected to migration assays, and **D** migrated cells were counted with Stat Monitor in Photoshop (mean ± SD, n = 3). ^*^P < 0.05, statistically significant difference vs. vector cells. ^#^*P* < 0.05 and ^^^*P* < 0.05, statistically significant difference vs. vector cells treated with Flag-mortalin

*RECK*, a tumor suppressor gene, is critical for the regulation of the migratory and invasive capacities of tumor cells [16]. In order to explore whether there is a regulatory effect of mortalin on the RECK/STAT3 signal pathway, we first transfected Flag-mortalin into HepG2 cells. As shown in Fig. 2A, mortalin overexpression significantly decreased the RECK protein level. At the same time, we found that the expression of RECK protein significantly increased after transfection of mortalin siRNA in HCCLM3 cells.

Studies have shown that RECK was an important molecule that regulates STAT3 [25]. STAT3 activation plays an important role in metastasis, and STAT3 increases the expressions of MMP2 and MMP9, which act as key mediators of the metastatic process of cancer cells. Na et al. found that JAK–STAT signaling was involved in mortalin-induced migration and invasion of breast cancer cells through analysis of the DNA microarray [14]. To explore the relationship between STAT3 and mortalin, we used mortalin siRNA or high expression plasmid, respectively, to knockdown or highly express mortalin. We found that the expression levels of p-STAT3 and Ac-STAT3 increased significantly after mortalin overexpression in HepG2, while the expression levels of p-STAT3 and Ac-STAT3 decreased after mortalin knockdown in HCCLM3 (Fig. 2A). In order to further explore the role of STAT3 in cell migration regulated by mortalin, we first used siRNA to knockdown STAT3 in HepG2 cells, and then conducted Transwell experiments. We found that the cell migration ability decreased significantly after knocking-down STAT3 (Additional file 1: Fig. S1). Then we observed the changes in EMT and cell migration after adding the p-STAT3 or Ac-STAT3 inhibitors STATTIC and C646 to HepG2 cells with high mortalin expression. We found that E-cadherin was down-regulated and N-cadherin and vimentin were up-regulated after high mortalin expression, and the EMT process was reversed after adding the inhibitors, and the up-regulation of migration induced by mortalin was also inhibited (Fig. 2B–D). These results confirmed that mortalin, through the RECK/STAT3 signaling pathway, promoted the migration of hepatocellular carcinoma cells.

### Mortalin is a specific target of salvianolic acid B

**Fig. 3:**
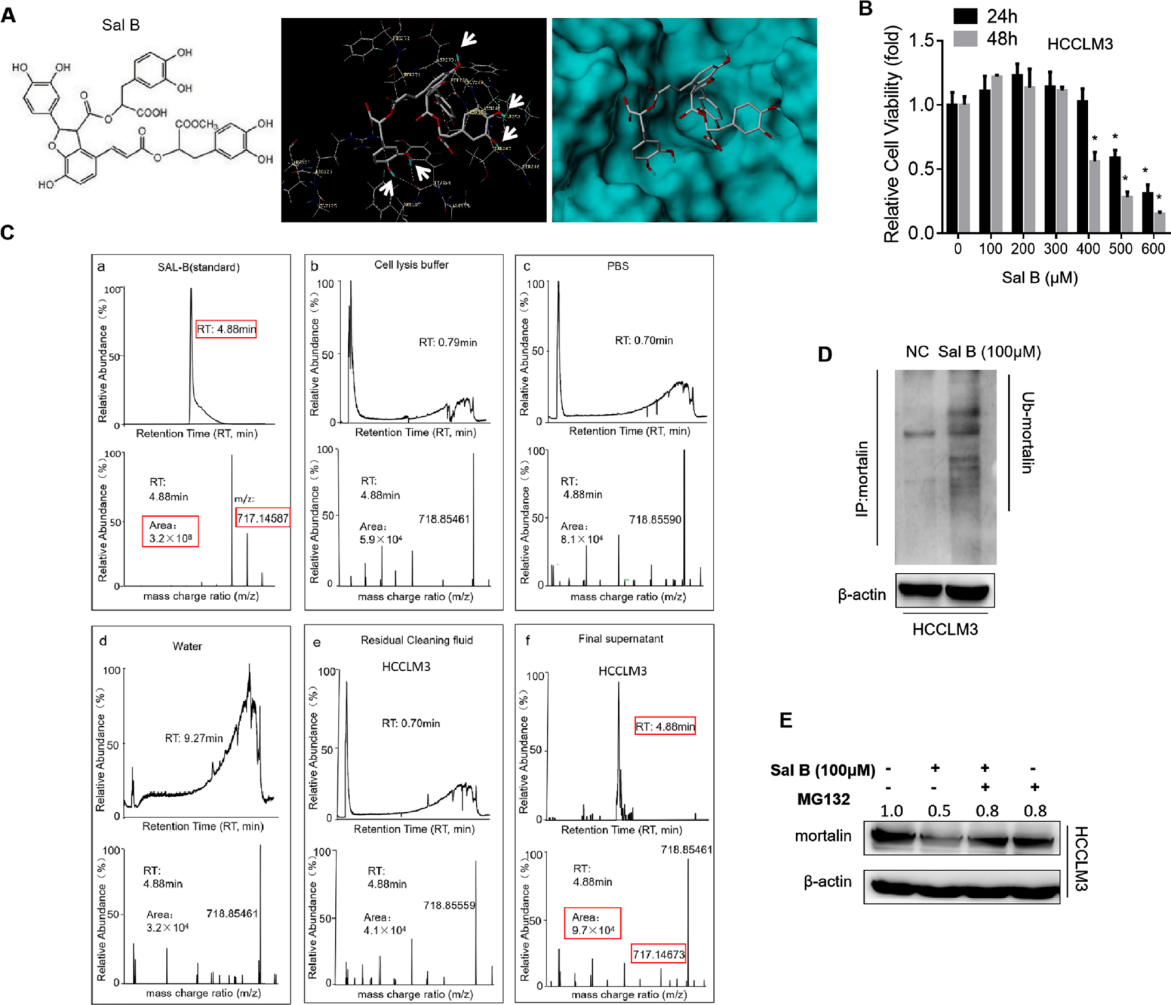
Mortalin is degraded by salvianolic acid B, a caffeic acid phenethyl ester analog, which is found to combine with mortalin. **A** Prediction of the binding of Sal B and mortalin by SYBYL-X software. **B** HCCLM3 cells were treated with 0–600 μM Sal B for 24 h or 48 h. Cell viability of the HCCLM3 cells was measured, *P < 0.05, statistically significant difference vs. 0 μM Sal B group. **C** 10 μM Sal B was used as a positive control and sterilized double-steamed water as a negative control. The retention time of Sal B was 4.88 min, the accurate mass number m/z was 717.1459, and the area was 9.7 × 10^4^. Red marker represents the detection of Sal B samples. **D** After treatment with Sal B, cells were harvested for ubiquitination analysis of mortalin by immunoprecipitation. **E** Western blots of mortalin expression after pretreatment with MG132. After pretreatment with 20 μM proteasome inhibitor MG132 for 2 h, HCCLM3 cells were treated with Sal B for 48 h, and then cells were harvested for western blot analysis

We initially predicted the binding of caffeic acid and its derivatives to mortalin protein using the molecular docking software SYBYL-X. We used three combinations to predict the interactions of caffeic acid and its derivatives with mortalin. The results showed that the binding score of Sal B was the highest among the three binding modes, indicating that the binding ability of Sal B to mortalin was the strongest. Then we chose Sal B for further software analysis. As shown in Fig. 3A, Sal B contains four benzene rings with hydroxyl groups attached to them. Two hydroxyl groups formed two hydrogen bonds with Tyr196 and Phe197. Another hydroxyl group formed one hydrogen bond with Asp270 and the other two hydroxyl groups formed two hydrogen bonds with Ile252 and Thr267, thus forming a stable bond (shown by the white arrow). Due to steric hindrance, Sal B was located in a cavity in mortalin that is located between residues 253 and 282 in the binding region of mortalin and p53. The classical inhibitor of mortalin, MKT-077, also bound to mortalin in this region [23]. In order to further confirm the interaction between Sal B and mortalin in the cells, we designed an immunoprecipitation-ultra-high performance liquid chromatography-mass spectrometry technique based on classical experiments to confirm the specific binding of Sal B with mortalin. As shown in Fig. 3B and Additional file 1: Fig. S3, Sal B had no effect on HCC cell viability at concentrations of 0.0, 100.0, 200.0, and 300.0 μM for 24 and 48 h; the cell samples had a Sal B concentration of 100.0 μM for 24 h. At the same time, we used a Sal B concentration of 10 μM as a positive control and sterilized double-steamed water as a negative control. Other confounding factors such as PBS, cell lysate, and the last PBS cleaning solution were also tested. The results showed that the retention time of Sal B was 4.88 min, the accurate mass number m/z was 717.1459 in the negative ion mode and the area was 9.7 × 10^4^. Sal B was detected in the cell samples, while Sal B was not detected in the other samples (Fig. 3C).

This suggested that Sal B bound mortalin specifically when other confounding factors were excluded from the immunoprecipitation process. However, it was not clear whether Sal B affected mortalin expression. Current studies have shown that ubiquitin-like protein UBXN2A promotes hydroxyl-terminal-dependent mortalin ubiquitination of HSP70 interacting protein (CHIP), and UBXN2A can increase mortalin proteasome degradation [18]. Therefore, we speculated whether Sal B degrades mortalin protein through ubiquitination. After immunoprecipitation of HCCLM3 cells treated with Sal B and mortalin antibody, we found that the ubiquitination level of mortalin in the cells treated with Sal B was significantly higher than that in the control group (Fig. 3D). And Sal B could significantly reduce mortalin protein expression, but this regulation was significantly inhibited in cells pretreated with MG132, a proteasome inhibitor (Fig. 3E). These results indicated that Sal B could specifically bind to mortalin, and increased the degradation of mortalin proteasomes through ubiquitination, thus inhibiting the expression of mortalin protein.

### Salvianolic acid B inhibits the migration and invasion of HCC via the RECK/STAT3 pathway

**Fig. 4:**
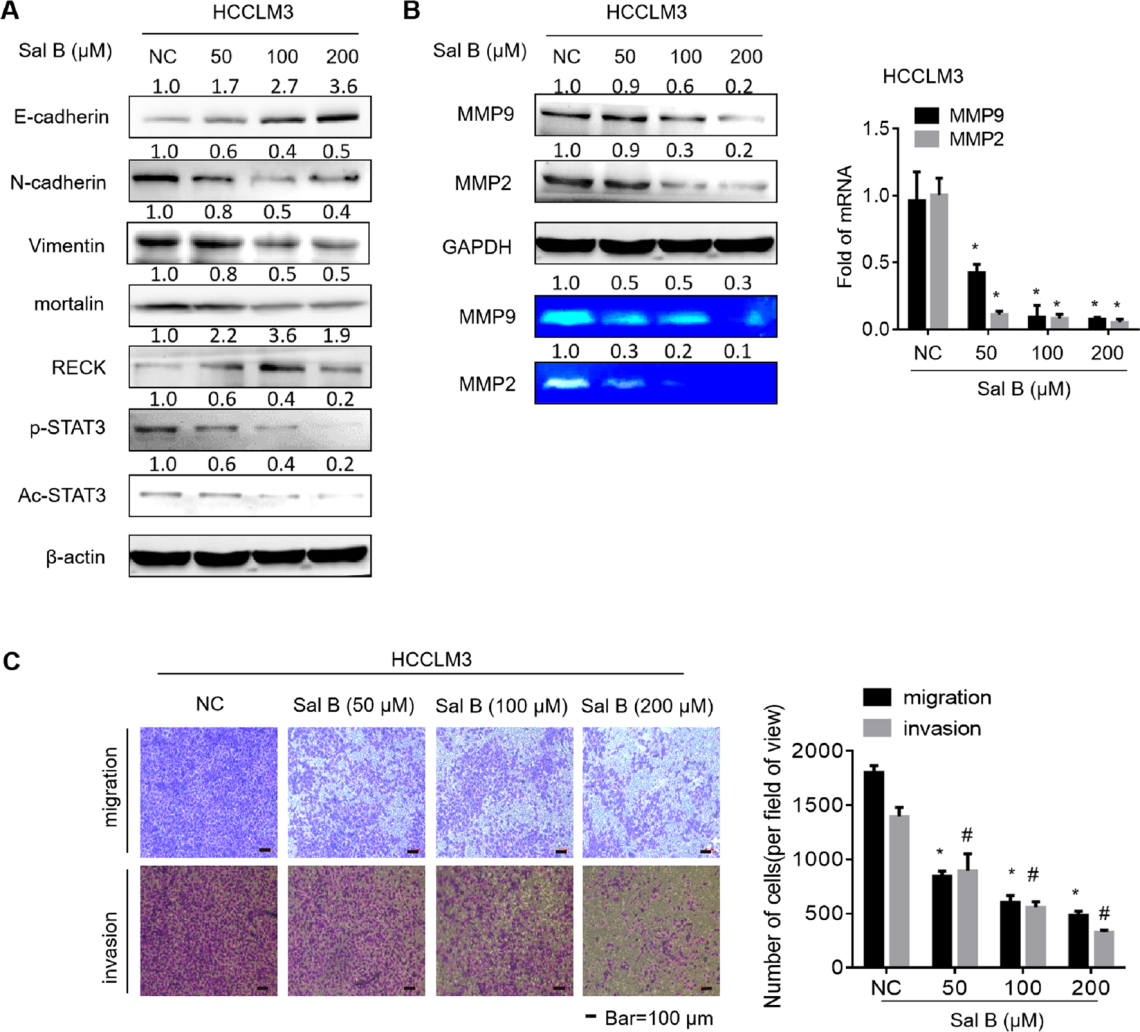
Salvianolic acid B can inhibit the migration and invasion of hepatocellular carcinoma cells. **A** Western blot assays of E-cadherin, vimentin, N-cadherin, mortalin, RECK, p-STAT3, Ac-STAT3, and β-Actin in HCCLM3 cells were performed. **B** Western blot assays of MMP2, MMP9, and GAPDH were performed. MMP2 and MMP9 activities in HCCLM3 were investigated by gelatin zymography assays. The mRNA levels of MMP2 and MMP9 were investigated by quantitative real-time polymerase chain reaction (qRT-PCR) analysis (mean ± SD, n = 3). ^*^P < 0.05, statistically significant difference vs. untreated cells. **C** HCCLM3 cells were subjected to migration and invasion assays, and migrated and invaded cells were counted with Stat Monitor in Photoshop (mean ± SD, n = 3). ^*^P < 0.05, ^#^P < 0.05, statistically significant difference vs. untreated cells. Bars = 100 μm

Considering the important link between the EMT/RECK–STAT3 signaling pathway/MMPs and tumor cell migration/invasion, HCCLM3 cells were treated with Sal B at 0.0, 50.0, 100.0, and 200.0 μM for 48 h, and the expressions of EMT-related proteins, including E-cadherin, N-cadherin, and vimentin, were analyzed. Sal B up-regulated the expression of E-cadherin and down-regulated the expressions of N-cadherin and vimentin, suggesting that Sal B could reverse the EMT process. At the same time, the protein levels of RECK increased and the protein levels of mortalin decreased in response to treatment of HCCLM3 with Sal B at different concentrations up to 200.0 μΜ for 48 h, and Sal B efficiently decreased the expressions of p-STAT3^Y705^ and Ac-STAT3^K685^ in HCCLM3 (Fig. 4A). Furthermore, Sal B down-regulated the expressions of MMP2 and MMP9. Gelatin zymography analysis demonstrated that Sal B inhibited the activities of MMP2 and MMP9 (Fig. 4B). In the scratch assay, 50.0, 100.0, and 200.0 μM Sal B inhibited the scratch healing abilities of HCC cells in a dose-dependent manner (Additional file 1: Fig. S2). As shown in Fig. 4C, Sal B decreased the migratory and invasive potential of HCCLM3 cells in a dose-dependent manner. These data indicated that Sal B inhibited the EMT, RECK/STAT3 signaling pathway and MMPs activities of HCCLM3, and attenuated the migratory and invasive abilities of HCCLM3.

### Salvianolic acid B inhibits the migration and invasion of HCC cells by regulating mortalin

As we have shown in the previous sections, mortalin promoted the migration and invasion of hepatocellular carcinoma cells via RECK/STAT3 signaling, and Sal B inhibited EMT, the RECK/STAT3 pathway, MMP9/MMP2, and the migration and invasion of hepatocellular carcinoma cells. As we found that Sal B specifically bound to mortalin and degraded it, we hypothesized that Sal B inhibited the migration and invasion of hepatocellular carcinoma cells stimulated by mortalin.

**Fig. 5:**
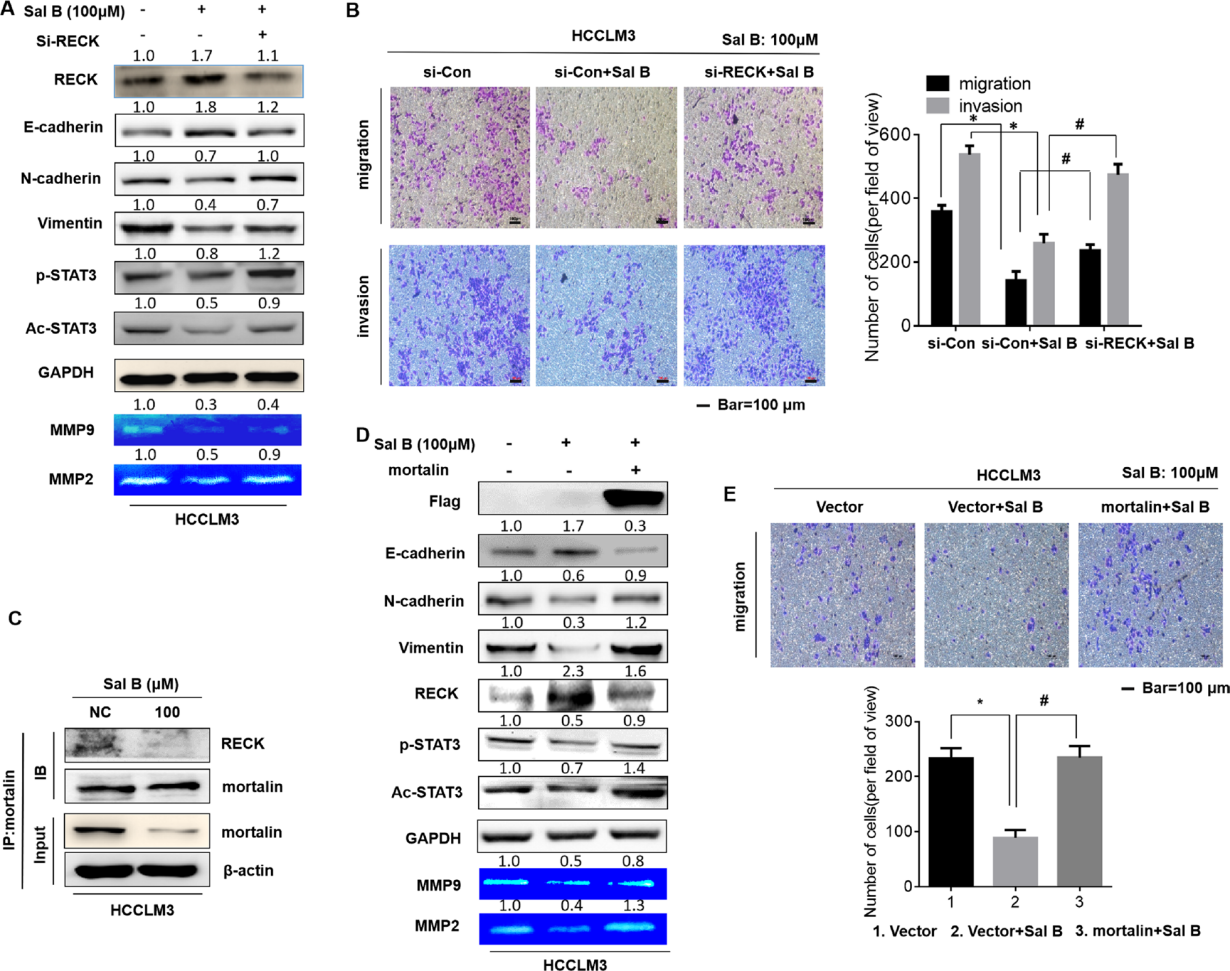
Salvianolic acid B inhibits the migration and invasion of hepatocellular carcinoma cells by regulating mortalin. **A** Western blot assays of E-cadherin, N-cadherin, vimentin, p-STAT3, Ac-STAT3, and GAPDH were performed. MMP2 and MMP9 activities in HCCLM3 cells were investigated by gelatin zymography assays. **B** HCCLM3 cells were subjected to migration and invasion assays, and migrated and invaded cells were counted with Stat Monitor in Photoshop (mean ± SD, n = 3). ^*^P < 0.05, statistically significant difference vs. si-NC cells. ^#^*P* < 0.05, statistically significant difference vs. si-NC cells treated with 100 μM Sal B. **C** After HCCLM3 cells were treated with NC or 100 μM Sal B for 48 h, the binding of mortalin to RECK was detected by immunoprecipitation. **D** HCCLM3 cells were transiently transfected with vector or Flag-mortalin for 8 h, and then the cells treated with 0 or 100 μM Sal B were incubated for an additional 24 h in fresh medium with 10% FBS. Western blot assays of E-cadherin, N-cadherin, vimentin, Flag, RECK, p-STAT3, Ac-STAT3, and GAPDH were performed. MMP2 and MMP9 activities were investigated by gelatin zymography assays. **E** HCCLM3 cells were subjected to the migration assays, and migrated cells were counted with Stat Monitor in Photoshop (mean ± SD, n = 3). ^*^P < 0.05, statistically significant difference vs. untreated cells. Bars = 100 μm. ^#^*P* < 0.05, statistically significant difference vs. si-NC cells treated with 100 μM Sal B

As shown in Fig. 5A, siRNA-mediated silencing of RECK was conducted to determine the effect of RECK on EMT markers and STAT3/MMP signaling in HCC cells. Cells were transiently transfected with control or RECK siRNA for 8 h, then exposed to 0 or 100 μΜ Sal B for 48 h. As shown in Fig. 5A, knockdown of RECK significantly reduced the Sal B-induced up-regulation of E-cadherin and down-regulation of N-cadherin and vimentin. In addition, we determined the role of RECK in the STAT3/MMP axis. Sal B down-regulated p-STAT3^Y705^ and Ac-STAT3^K685^, and knockdown of RECK abolished the Sal B-induced down-regulation of p-STAT3^Y705^ and Ac-STAT3^K685^. Gelatin zymography analysis demonstrated that knockdown of RECK significantly enhanced the Sal B-induced repression of MMP2 and MMP9. Collectively, these results suggested that Sal B modulated EMT markers and STAT3/MMP signaling in HCC cells by up-regulating RECK. Based on the results described above, we hypothesized that RECK was involved in the Sal B-induced inhibition of migration and invasion of HCC cells. To verify this hypothesis, we treated HCCLM3 cells subjected to RECK knockdown with Sal B to determine their migratory/invasive abilities. As shown in Fig. 5B, knockdown of RECK blocked the Sal B-induced inhibition of the migratory and invasive abilities of HCC cells. These results suggested that RECK was involved in the anti-metastatic effects of Sal B in HCC cells. The immune coprecipitation assay showed that mortalin bound to RECK, and this bonding was weakened by Sal B (Fig. 5C).

To further confirm the effect of mortalin on Sal B inhibition of the migration and invasion of HCC cells, we overexpressed mortalin on the basis of Sal B treatment. As shown in Fig. 5D, high expression of mortalin combined with Sal B significantly reduced the up-regulation of E-cadherin induced by Sal B, and reversed the down-regulation of N-cadherin and vimentin induced by Sal B. At the same time, Sal B down-regulated p-STAT3^Y705^ and Ac-STAT3^K685^, and high expression of mortalin abolished the Sal B-induced down-regulation of p-STAT3^Y705^ and Ac-STAT3^K685^_._ In addition, gelatin zymograms also confirmed that high expression of mortalin combined with Sal B significantly reversed the decrease in MMP2/MMP9 regulated by Sal B (Fig. 5D). Transwell experiments also confirmed that high expression of mortalin combined with Sal B could significantly reverse the migration ability of hepatocellular carcinoma cells inhibited by Sal B (Fig. 5E). These results suggested that mortalin was involved in the process of migration and invasion of HCC cells inhibited by Sal B.

## Conclusion

In summary, our results showed that mortalin played an important role in the migration and invasion of HCC cells, and the RECK/STAT3 signal pathway was regulated by mortalin. Furthermore, we found that salvianolic acid B, a caffeic acid phenethyl ester analog, specifically bound to mortalin and increased the degradation of mortalin proteasomes through ubiquitination, thereby up-regulating RECK, inhibiting STAT3, and finally inhibiting the migration and invasion of HCC cells. Our work suggested that mortalin is a potential therapeutic target for hepatocellular carcinoma.

## Discussion

HCC is a highly metastatic tumor that is associated with high recurrence rates and low survival rates [20]. The molecular mechanisms underlying the suppression of invasion and migration in HCC have been extensively studied to discover new therapeutic targets and predictive markers. The EMT is an important step in tumor migration and invasion, and it is characterized by the loss of the epithelial properties of cells, such as adhesion and the expression of the epithelial marker E-cadherin, and the acquisition of mesenchymal properties, such as increased cell motility and the up-regulation of the mesenchymal markers N-cadherin and vimentin [10].

The extracellular matrix (ECM) is an important tissue barrier for tumor metastasis. The migration and invasion of malignant tumors are often accompanied by changes in the expression of the ECM and its cell surface receptors [13]. The degradation of the ECM by MMPs is one of the key aspects of tumor cell metastasis. Many malignant tumors are associated with increased secretion and activity of MMPs [19].

**Fig. 6:**
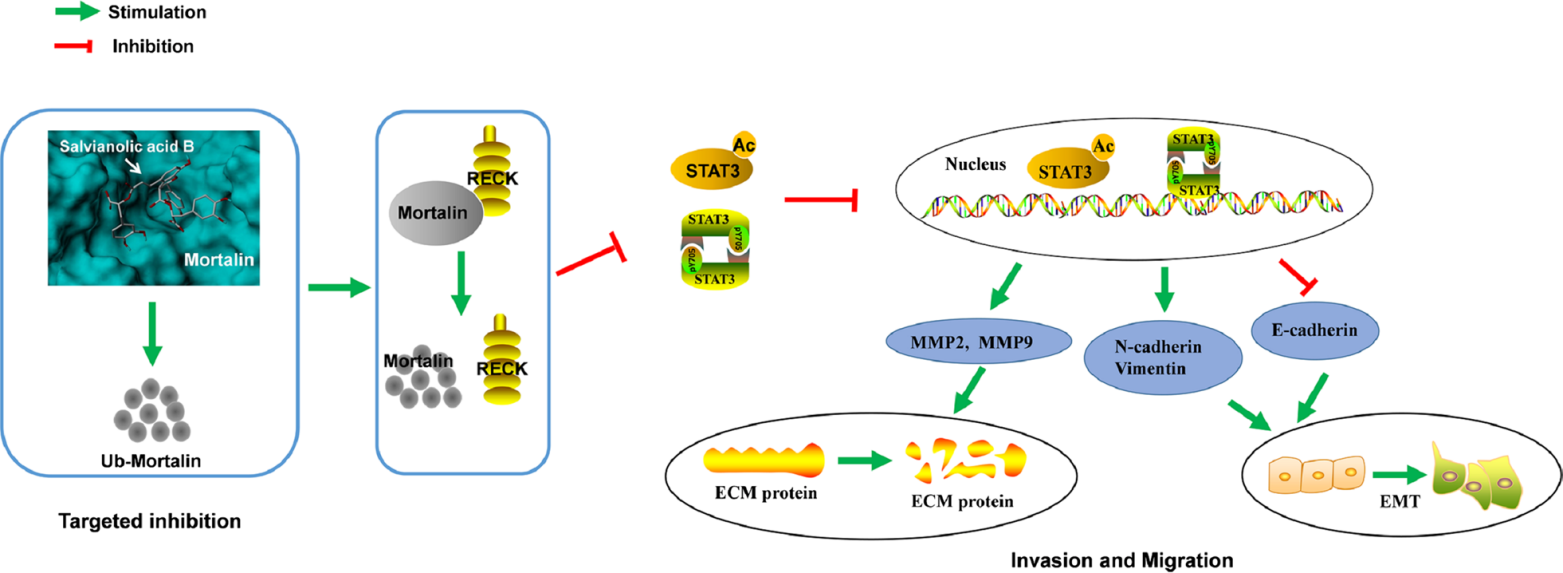
The signaling pathway was elucidated in hepatocellular carcinoma cells after Sal B treatment. Sal B can specifically bind to mortalin, and increased the degradation of mortalin proteasomes through ubiquitination. The down-regulation of mortalin can lead to the up-regulation of RECK protein. Then its downstream p-STAT3 and Ac-STAT3 were inhibited to enter the nucleus, resulting in a decrease of the invasive and migratory abilities of hepatocellular carcinoma cells

Mortalin, a member of the Hsp70 family, plays an important carcinogenic role in cancer cells through a variety of mechanisms. In addition, mortalin is considered a target for many kinds of cancer therapy [1, 7, 21, 24, 26]. It is therefore important to find specific mortalin inhibitors for the treatment of tumors. Current studies have shown that mortalin has good surface properties and can cause small molecules to dock with high affinity and specificity. Wadhwa et al. used the molecular docking software Autodock to predict that caffeic acid phenylethyl ester (CAPE) could dock with mortalin and prevent the formation of mortalin-p53 complex, resulting in nuclear ectopia of mortalin and activation of the p53 anti-cancer function, and inhibit the growth of cancer cells through the p53-GAD45a-p21 pathway [22]. Over-inhibition of matrix metalloproteinases inhibits cell invasion and metastasis. At the same time, CAPE can increase the sensitivity of anticancer drugs by targeting mortalin in many ways [28]. CAPE and Sal B investigated in this study are caffeic acid derivatives, so we speculated whether Sal B would also have the same effect, targeting mortalin to play an anti-cancer role. We also found that another specific inhibitor of mortalin, MKT-077, bound to mortalin in the same region, and MKT-077 inhibited its interaction with p53 without affecting mortalin expression, thus activating the anti-cancer function of p53 [4, 23]. As shown in Fig. 6, we found that Sal B could significantly bind to mortalin protein. We consider that maybe Sal B can be used as a new inhibitor of mortalin, and the further research to elucidate is still needed. How does Sal B affect mortalin expression? It has been reported that ubiquitin-like protein UBXN2A promoted the HSP70 interacting protein (CHIP) dependent ubiquitination of the carboxyl end of mortalin. Subsequently, it was found that UNXN2A increased mortalin proteasome degradation. Subcellular regionalization experiments showed that induction of UNXN2A reduced mortalin and its partner HSP60 levels. Up-regulation of UNXN2A by the small molecule veratridine (VTD) can decrease mortalin levels in cancer cells. Consistent with the results in vitro, UNXN2A^±^ mice showed increased mortalin selectivity in colon tissues. Recombinant UNXN2A can enhance the degradation of mortalin proteasomes in mouse colon tissue [18]. Our results also proved that Sal B degraded mortalin protein through ubiquitination.

Many studies have recently been conducted to investigate mortalin and the migration and invasion of tumors. It has been proved that mortalin can promote the migration and invasion of tumors, including breast cancer, liver cancer, cholangiocarcinoma, etc. [9, 14, 27]. However, the mechanism underlying its effect on the migration and invasion of tumors remains unclear. Our current experiments confirmed that mortalin affected the migration and invasion of HCC cells through the EMT and matrix metalloproteinase pathways. RECK and STAT3 are also involved as upstream signaling molecules. RECK is down-regulated in many tumors, and the mechanism of this down-regulation is multifactorial and tumor-specific. One of the common targets of inhibition is the Sp1 site in the promoter sequence of RECK. Early studies speculated that the oncogene Ras promotes the phosphorylation or other modification of Sp1/Sp3 factor by activating the extracellular signal-regulated kinase (ERK) pathway, increases the affinity of the Sp1 site on RECK promoter, and reduces the expression of RECK [16]. Other studies hypothesized that the interaction between histone deacetylase and Sp1 might help to inhibit RECK transcription [11]. Our study showed that Sal B could up-regulate the expression of RECK and down-regulate the expression of mortalin, and that mortalin could affect the protein level of RECK. It is not clear whether mortalin can affect RECK through the transcriptional regulation of Sp1 or in other ways, all of which need further research.

Overactivation of STAT3 is essential for metastasis. RECK regulates STAT3 activation, cytokine signaling, and induction of vascular endothelial growth factor and uPA by forming complexes with cell surface receptors [25]. MMP9 is secreted by paracrine cancer cells with high STAT3 signal transduction, and STAT3 can regulate MMP9 [17]. In addition, STAT3 blocked MMP2 expression and MMP9 promoter activity through the use of chromatin immunoprecipitation of the anti-STAT3 antibody [29]. We suggest that Sal B induces the up-regulation of RECK, which leads to the down-regulation of p-STAT3^Y705^ and Ac-STAT3^K685^, and inhibits the ability of MMPs to destroy the ECM, thus inhibiting the migration and invasion of HCC cells (Fig. 6). Yuki et al. showed that RECK was up-regulated after the EMT in non-malignant epithelial cells, but in cancer-derived cell lines, the down-regulation of E-cadherin and up-regulation of RECK were not related. Our current studies suggested that Sal B regulated EMT markers and STAT3/MMPs signal transduction in HCC cells by up-regulating RECK. All changes in EMT-related molecular markers knocked-down by RECK were based on Sal B treatment. Whether RECK can affect the EMT in HCC cells without Sal B remains to be further confirmed.

Na et al. showed that JAK–STAT signaling was involved in the mortalin-induced migration and invasion of breast cancer cells through DNA microarray analysis [14]. However, the relationship between mortalin and the STAT signaling pathway still lacks experimental evidence. This is the first time it has been shown that Sal B significantly reduced the levels of p-STAT3 and Ac-STAT3, mortalin regulated the levels of p-STAT3 and Ac-STAT3, and high expression of mortalin reversed the down-regulation of p-STAT3 and Ac-STAT3 by Sal B. However, research to further elucidate the mechanism is still needed.

## Supplementary Information

**Additional file 1.** Supplementary data of this article.

## Data Availability

Data available on request from the authors: The data that support the findings of this study are available from the corresponding author upon reasonable request.

## Abbreviations

Sal B: Salvianolic acid B
RECK: The reversion-inducing cysteine-rich protein with kazal motifs
MMPs: Max metalloproteinases
STAT3: Signal transducer and activator of transcription 3
HCC: Hepatocellular carcinoma
EMT: Epithelial–mesenchymal transition
ECM: Extracellular matrix
CAPE: Caffeic acid phenethyl ester
