# Supplementary material for: Salvianolic acid B targets mortalin and inhibits the migration and invasion of hepatocellular carcinoma via the RECK/STAT3 pathway
Source: Cancer Cell Int. 2021 Dec 7;21:654. doi: 10.1186/s12935-021-02367-z (PMC8650508; doi:10.1186/s12935-021-02367-z)
Supplement: Supplementary file 1 — Additional file 1. Supplementary data of this article. [file 12935_2021_2367_MOESM1_ESM.docx]

**Additional information**

**Title:** Salvianolic acid B specifically binds mortalin to attenuate the migration and invasion of hepatocellular carcinoma cells by inhibiting RECK/STAT3 signaling pathway

**Author list:** Mengying Teng*, Chunyan Hu*, Bingmo Yang，Wei Xiao, Qian Zhou, Yuan Li ^🖂^ and Zhong Li ^🖂^

**Inventory:**

1. Additional tables, page 2-4

2. Additional figures and their legends, pages 5-8

**1. Additional tables**

**1.1. Table S1. Primers used in this study.**

| Names | | Primers | |
| --- | --- | --- | --- |
| MMP2 | F:CCCACTGCGGTTTTCTCGAAT  R:CAAAGGGGTATCCATCGCCAT | |  |
| MMP9 | F:TGTACCGCTATGGTTACACTCG  R:GGCAGGGACAGTTGCTTCT | |  |
| β-actin | F:TCAGGTCATCACTATCGGCACT  R:AAAGAAAGGGTGTAAAACGCA | |  |

**1.2. Table S2. Antibodies used in this study.**

| Names | Web Link | Source | Used |
| --- | --- | --- | --- |
| p-STAT3 | <https://www.cellsignal.com/products/primary-antibodies/phospho-stat3-tyr705-d3a7-xp-rabbit-mab/9145?_=1473248676908> | Cell Signaling Technology | 1: 1000 |
| Ac-STAT3 | <https://www.cst-c.com.cn/products/primary-antibodies/acetyl-stat3-lys685-antibody/2523?site-search-type=Products> | Cell Signaling Technology | 1: 1000 |
| E-cadherin | <https://www.cellsignal.com/products/primary-antibodies/e-cadherin-24e10-rabbit-mab/3195?N=4294956287&Ntt=24E10&fromPage=plp> | Cell Signaling Technology | 1: 1000 |
| N-cadherin | <https://www.cellsignal.com/products/primary-antibodies/n-cadherin-d4r1h-xp-rabbit-mab/13116?site-search-type=Products&N=4294956287&Ntt=n-cadherin&fromPage=plp> | Cell Signaling Technology | 1: 1000 |
| Vimentin | <https://www.cellsignal.com/products/primary-antibodies/vimentin-d21h3-xp-rabbit-mab/5741?N=4294956287&Ntt=vimentin&fromPage=plp> | Cell Signaling Technology | 1: 1000 |
| MMP2 | <https://www.cst-c.com.cn/products/primary-antibodies/mmp-2-d4m2n-rabbit-mab/40994?site-search-type=Products&N=4294956287&Ntt=mmp2&fromPage=plp> | Cell Signaling Technology | 1: 1000 |
| MMP9 | <https://www.cst-c.com.cn/products/primary-antibodies/mmp-9-d6o3h-xp-rabbit-mab/13667?site-search-type=Products&N=4294956287&Ntt=mmp9&fromPage=plp> | Cell Signaling Technology | 1: 1000 |
| RECK | <https://www.cst-c.com.cn/products/primary-antibodies/reck-d8c7-rabbit-mab/3433?site-search-type=Products&N=4294956287&Ntt=reck&fromPage=plp&_requestid=64915> | Cell Signaling Technology | 1: 1000 |
| Mortalin | <https://www.cst-c.com.cn/products/primary-antibodies/grp75-d13h4-xp-rabbit-mab/3593?site-search-type=Products&N=4294956287&Ntt=mortalin&fromPage=plp> | Cell Signaling Technology | 1: 1000 |
| Flag | http://www.affbiotech.cn/goods-6269-T0003-Flag-Tag+Antibody.html | Affinity | 1:1000 |
| Ubiquitin | <https://www.ptgcn.com/products/ubiquitin-Antibody-10201-2-AP.htm> | Proteintech Group | 1: 1000 |
| β-Actin | <http://www.beyotime.com/product/AA128.htm> | Beyotime | 1:1000 |
| GAPDH | <http://www.beyotime.com/product/AG019.htm> | Beyotime | 1:1000 |

**1.3. Table S3. siRNAs used in this study.**

| Names | Web Link | Source | Used |
| --- | --- | --- | --- |
| RECK-  siRNA | <https://datasheets.scbt.com/sc-39718.pdf> | Santa Cruz Biotechnology | 50 nM |
| Mortalin-  siRNA | <https://datasheets.scbt.com/sc-35520.pdf> | Santa Cruz Biotechnology | 50 nM |
| STAT3-  siRNA | https://datasheets.scbt.com/sc-29493.pdf | Santa Cruz Biotechnology | 50 nM |
| NC-  siRNA | <http://datasheets.scbt.com/sc-37007.pdf> | Santa Cruz Biotechnology | 50 nM |

**2. Additional Figures and their legends**

2.1. Figure S1


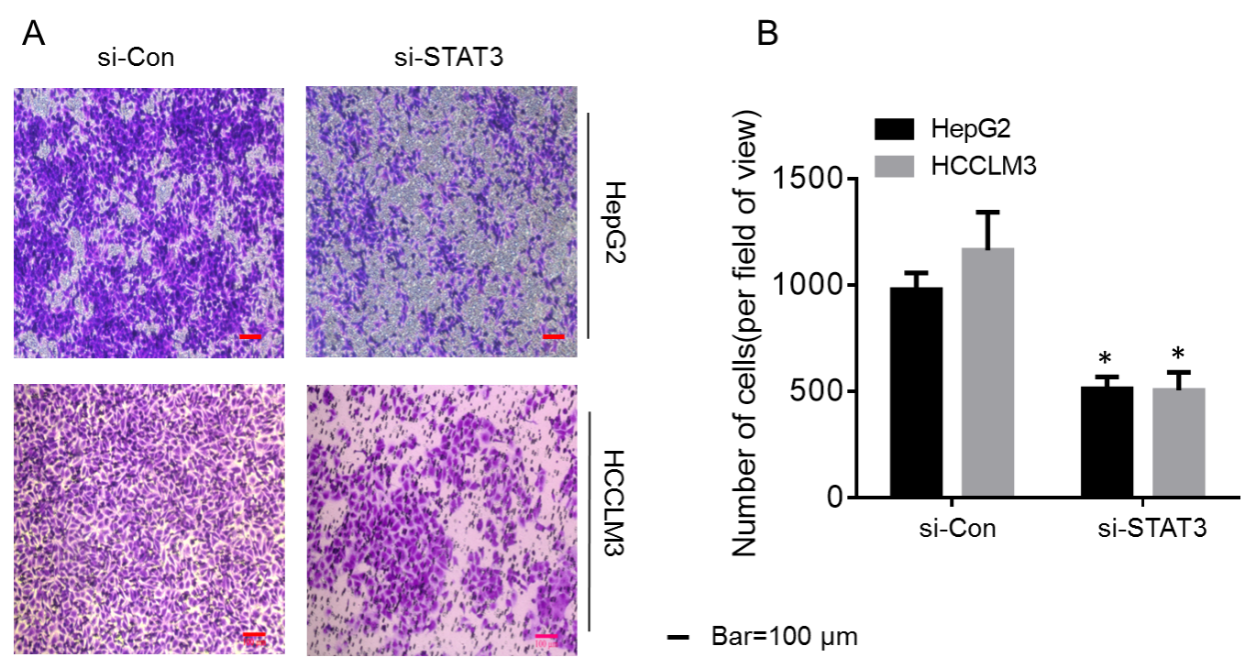


2.1. Figure S1. Effect of STAT3 in migration of hepatocellular carcinoma cells. HepG2 and HCCLM3 cells were subjected to the migration assays with different STAT3 levels, and migrated cells were counted with Stat Monitor in photoshop (mean ± SD, n=3). *P < 0.05, statistically significant difference vs. untreated cells (A and B). Bars = 100 μm.

2.2. Figure S2


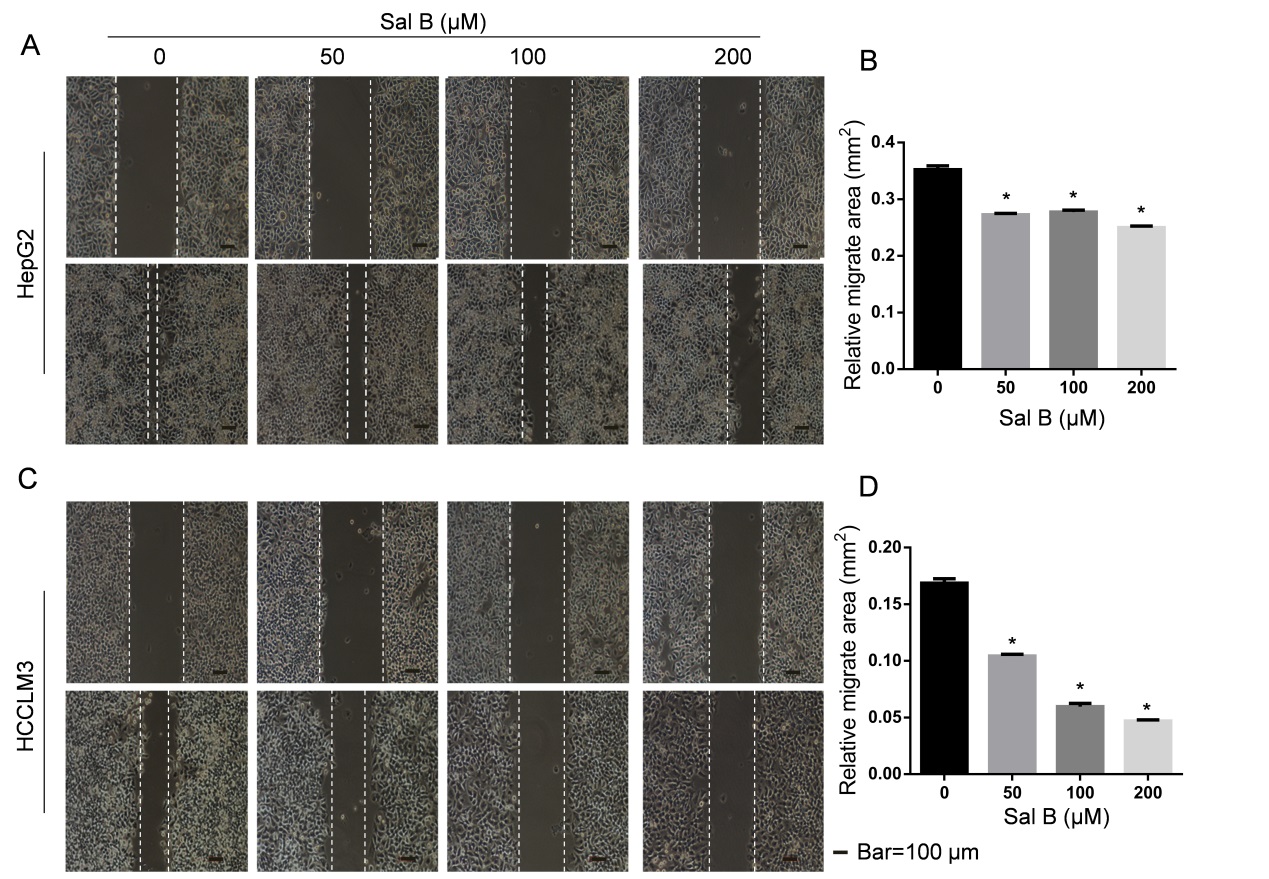


2.2. Figure S2. Sal B inhibited the scratch healing process of hepatocellular carcinoma cells. (A and B) HepG2 cells were exposed to 0.0, 50.0, 100.0, or 200.0 μM Sal B for 48 h, Wound healing assay analyses were performed, relative levels of cell migration areas were determined by Image J. Bars=100μm. (C and D) HCCLM3 cells were exposed to 0.0, 50.0, 100.0, or 200.0 μM Sal B for 24 h, Wound healing assay analyses were performed, relative levels of cell migration areas were determined by Image J. Bars=100μm.

2.3. Figure S3

*****

*****

*****

*****

*****

*****

HepG2

Sal B (μM)

2.3. Figure S3. HepG2 cells were treated with 0- 600 μM Sal B for 24h or 48 h. Cell viability of HepG2 cells was measured, **P* < 0.05, statistically significant difference vs. 0 μM Sal B group.

2.4. Figure S4

2.4. Figure S4. (A) HepG2 cells were transiently transfected with vector or Flag-mortalin for 8 h. The mRNA levels of mortalin was investigated by quantitative real-time polymerase chain reaction (qRT-PCR) analysis (mean ± SD, n=3) . **P* < 0.05, statistically significant difference vs. untreated cells.(B) HCCLM3 cells were transiently transfected with vector or siRNA-mortalin (1,2,3) for 8 h. The mRNA levels of mortalin was investigated by quantitative real-time polymerase chain reaction (qRT-PCR) analysis (mean ± SD, n=3) . **P* < 0.05, statistically significant difference vs. untreated cells.
